# Comment on the “Ground Water Chemistry Changes before Major Earthquakes and Possible Effects on Animals”, by R. A. Grant, T. Halliday, W. P. Balderer, F. Leuenberger, M. Newcomer, G. Cyr and F. T. Freund. *Int. J. Environ. Res. Public Health*, 2011, *8*, 1936–1956

**DOI:** 10.3390/ijerph9072339

**Published:** 2012-07-02

**Authors:** Vassiliki Katsika-Tsigourakou

**Affiliations:** Department of Solid State Physics, Faculty of Physics, University of Athens, Panepistimiopolis, 15784 Zografos, Greece; Email: vkatsik@phys.uoa.gr; Tel.: +30-210-727-6813; Fax: +30-210-727-6711

**Keywords:** groundwater chemistry, anomalous animal behavior, electromagnetic fields, precursors

## Abstract

Here, we suggest that electromagnetic emissions before rupture may be the mechanism for the explanation of abnormal behavior of animals before earthquakes.

## 1. Introduction

We share the opinion of the authors of Ref. [1] that many changes in the environment prior to major earthquakes have been documented. Actually these changes have been observed on the land surface, in water, in the air and in the ionosphere. Some of these changes, and in particular the electric and magnetic field precursory changes, have been used in practice in the 1990s [2,3] and 2000s [4] to announce the parameters (epicenter, magnitude and time-window) of impending major earthquakes in Greece.

The authors of Ref. [1] suggest that the key to understanding these diverse pre-earthquake phenomena has been the discovery that, when tectonic stesses build up in the Earth’s crust, highly mobile electronic change carries are activated. These carries, which are positive holes, can flow out of the stressed rock volume and spread into the surrounding unstressed rocks. When these positive holes arrive at the earth’s surface, they cause a range of follow-on reactions resulting in air ionization, injecting massive amounts of primarily positive air ions into the lower atmosphere. When they arrive at the rock-water interface, they act as O radicals, oxidizing water to hydrogen peroxide. In addition, other reactions take place which include the oxidation or partial oxidation of dissolved organic compounds. The authors of Ref. [1] suggest that some of compounds thus formed, may be irritants or toxins to certain species of animals, which are possibly the origin of their anomalous behavior before major earthquakes.

In the above frame, the authors of Ref. [1] explain the unusual behavior of common toads, *Bufo bufo* before the devastating earthquake of magnitude (M) 6.3 at L’ Aquila, Italy that occurred on 6 April 2009. In particular, a few days before this destructive event Grant and Halliday reported [5] that the toads disappeared from their breeding site (in a small lake located around 75 Km from the epicenter) and returned after the aftershock series. In short, potential changes in groundwater chemistry prior to seismic events and their possible effects on animals are discussed in Ref. [1]. 

Here, we point out that an alternative point of view has been published several months before the occurrence of L’ Aquila earthquake. In particular, Philippetis [6], after reviewing the mechanisms through which electromagnetic radiation can markedly influence living organisms, deduced that the most likely candidate is the recent mechanism proposed by Panagopoulos *et al.* [7], which foresees that low frequency (*i.e.*, of the order of 1 KHz or lower) external electric fields of amplitude of a few V/m can cause significant biological effects. Then Philippetis, by considering that such low frequency electric field variations have actually been detected (e.g., see refs [8,9,10,11]) before major earthquakes, finally concluded that the latter electric variations (which of cource are accompanied by magnetic field variations) constitute the origin for the anomalous biological effects observed before earthquakes, thus providing an explanation for the abnormal preseismic behavior of some animals. Philippetis’ suggestion seems to be strengthened by the following fact: Low frequency electric signals were actually detected before L’ Aquila earthquake, which occurred several months after the publication of Ref. [6]. In particular, it has been reported [12,13] that in a broad frequency range, well documented electromagnetic emission have been detected some days in advance, *i.e.*, on 26 March 2009 and on 4 April 2009, respectively.

The analysis presented by Grant *et al.* [1] approaches the coincidence of unusual toad behavior and precursory ionospheric disturbances from a different perspective. Both phenomena are driven by a physical process in the Earth crust, in the future focal volume, by the activation of h^*^ charge carriers (a process advanced by Freund and coworkers [14,15,16] usually termed peroxy defects model) during the rapid increase in tectonic stress prior to the earthquake. As h^*^ charge carriers spread out, they cause different secondary processes at the land surface and at the rock-water interface. It is the process at the rock-water interface consisting of changes in the ground-water and presumably lake water chemistry, which seems to have provided the toads with an impetus to leave and seek refuse on higher ground. Thus, it seems that the peroxy defects model [14,15,16] provides a consistent framework for the explanation of both the toads unusual behavior and the generation of the EM precursory phenomena, the latter being attributed to the generation of electric currents. We note that such a generation of electric currents basically agrees with the pressure-stimulated currents model advanced in Ref. [17] (and further treated in Ref. [18]) which suggests that a co-operative orientation of the electric dipoles (formed due to point defects) occurs when the stress in the future focal area reaches a critical value. This is in agreement with studies [19,20] showing that precursory electric signals have a scale-invariant structure which is a hallmark of criticality. 

Finally, we just note that the electrical measurements in Ref. [21] at a site lying only a few tens of Km from the epicenter of L’Aquila earthquake, which showed electrical oscillations of amplitude of the order micro-volts/meter in the range of hundreds of Hz (not being sufficient according to the work of Ref. [7] to enable the passage of ions across the cell membrane), do not preclude the alternative possibility we point out here in view of the following: First, the aforementioned monitoring site may not constitute (due to the electrical geoelectrical structure in the area) a station appreciably sensitive to the recording of precursory Seismic Electric Signals as advocated in Refs [2] and [3]. Note that the SES sensitivity may greatly vary among neighboring sites, e.g., see the different response to the sites A, B, C (differing by no more than 1 km) in Ioannina station in Northwestern Greece to the SES recordings as reported in a series of publications Refs [22,23,24,25,26]. Furthermore, note that the frequency range of the SES activities is ≤1 Hz which does not overlap with that in Ref. [21], in which “signatures have revealed horizontally oriented electric fields, between 20 Hz to 400 Hz (lasting from several minutes to up to two hours)”. Second, concerning the possible consequences of the presence of electric fields in the ELF band on the behavior of organisms, there are no yet studies that connect the possibility of the passage of ions into the cell membranes.
